# Immersive Virtual Environment Technology to Supplement Environmental Perception, Preference and Behavior Research: A Review with Applications

**DOI:** 10.3390/ijerph120911486

**Published:** 2015-09-11

**Authors:** Jordan W. Smith

**Affiliations:** Center for Geospatial Analytics, NC State University, Raleigh, NC 27695, USA; E-Mail: jordan_smith@ncsu.edu; Tel.: +1-435-830-6294; Fax: +1-435-797-4048

**Keywords:** ecological validity, experimental control, virtual reality, experimental research

## Abstract

Immersive virtual environment (IVE) technology offers a wide range of potential benefits to research focused on understanding how individuals perceive and respond to built and natural environments. In an effort to broaden awareness and use of IVE technology in perception, preference and behavior research, this review paper describes how IVE technology can be used to complement more traditional methods commonly applied in public health research. The paper also describes a relatively simple workflow for creating and displaying 360° virtual environments of built and natural settings and presents two freely-available and customizable applications that scientists from a variety of disciplines, including public health, can use to advance their research into human preferences, perceptions and behaviors related to built and natural settings.

## 1. Introduction

Research on how individuals’ values, beliefs, attitudes and behaviors are influenced by built and natural settings is inherently interdisciplinary and has used a variety of research methodologies. Participant observation, social surveys and laboratory experiments are widely used and indispensible tools. However, the ability of each of these methodologies to accurately represent and acutely control environmental stimuli varies widely, often leaving researchers unable to conclusively reject all possible confounding factors affecting their hypotheses. In response, a number of social scientists have become interested in the use of immersive virtual environment (IVE) technology as a complementary methodological tool given its capabilities of representing and controlling environmental stimuli [1,2]. The objectives of this paper are three fold. The first objective is to describe how IVE technology can be used to complement traditional methodologies used to investigate how individuals perceive and respond to built and natural environments; emphasis is placed on potential applications to public health research. The second objective of this paper is to describe a relatively simple workflow for creating and displaying 360° virtual environments of built and natural settings. The final objective is to present two editable and customizable applications that scientists from a variety of disciplines, including public health, can use to advance their research into human preferences, perceptions and behaviors related to built and natural settings.

## 2. IVE Technology 

Virtual environments are “synthetic sensory information that leads to perceptions of environments and their contents as if they were not synthetic” [1]. IVEs in turn, are ones that ‘surround’ an individual and create the perception they are enclosed within and interacting with environments that provide a continuous stream of stimuli [3]. IVEs are created through the integration of various hardware and software systems [1,4]. All systems include a user interface displaying the virtual environment to users, a tracking system recording the users’ movements and a computer that selects appropriate portions of the virtual environment to be displayed within the interface. IVE technologies provide a flexible and low-cost option for both creating and displaying virtual environments to research subjects (the IVE system and workflow described in this paper can be fully implemented for under $3000 USD).

IVEs can be experienced simultaneously by groups or by single individuals. Virtual environments can be projected to groups from behind using translucent screens surrounding the group in a cube-like environment [5] or via front projection systems. Front projection systems, which are more frequently used, can vary widely from semi-immersive environments, such as curved wall displays, to fully-immersive dome environments [6,7,8,9,10]. Group immersion can also be accomplished through arrays of flat screen displays. Across these different group display types, all users wear 3D glasses while a primary user also wears one or more positioning devices which tracks their body movements; the virtual environment is subsequently rendered according to the primary user’s position. While group IVE systems can be used in psychology and social psychology research, they have primarily been used as a novel medium through which educational material are presented [11].

As an alternative to group IVEs, virtual environments can be presented to single users, such as study participants, via head-mounted display systems. Head-mounted displays present virtual environments via single or multiple displays placed immediately in front of a research participant’s eyes; the intent is to occupy as much of the participant’s field-of-view as possible with the display. An essential component of presenting IVEs via head-mounted displays is tracking users’ head movements. Today, most head-mounted display devices accomplish this via sophisticated inertial tracking systems. Inertial and video tracking systems are also capable of monitoring the position and orientation of users’ hands or even their whole body by wearing specially designed gloves or suits and using software capable of dynamically rendering corresponding virtual objects (e.g., the hands or body positions of avatars). Head-mounted displays have historically been out of the reach of social scientists because of their high costs plus the installation expenses of additional graphics cards required for the simultaneous presentation of images in multiple displays. Advances in the standard offerings of computer hardware systems as well as the large gaming market have drastically improved access. Head-mounted display systems are now commercially available at costs well within even student budgets.

IVEs are capable of displaying and manipulating visual (sight), auditory (hearing), gustatory (taste), haptic (touch), olfactory (smell) or thermal (temperature) sensory stimuli. The reproduction of recorded three-dimensional sound-fields has been explored for over three decades [12,13,14,15,16]. Haptic stimuli, while being used relatively less than auditory stimuli, is already being applied to questions in environmental psychology such as the study of individuals’ environmentally responsible behavior [17]. Olfactory and thermal stimuli are infrequently used in research using IVE technology; however, some empirical evidence suggests they can play important roles in perception, cognition and memory [18,19,20]. The array of directly controllable stimuli provides virtual environment developers the ability to present immersive environments with varying degrees of realism, or ‘presence’ as it is commonly referred [21]. This provides the opportunity for researchers to examine the effects of various stimuli’s existence and magnitude on individuals’ perceptions, preferences and behaviors. However, almost all previous research utilizing IVE technology has used fully synthetic virtual environments. The field of psychophysics for example, is focused entirely on understanding psychological responses to virtual stimuli that represent physical objects. While this line of research has led to many nuanced developments in understanding of how visual cues and image quality affect sensory and perceptual responses, the use of fully synthetic virtual environments has limited adoption of the technology by investigators with more applied research agendas. There is a differentiation between IVE research based on wholly synthetic environments and research focused on replicating natural environments; the workflow presented below focuses on the latter. 

IVE technology provides scientists with the capacity to collect an array of response data varying in ontological complexity. Low-level reflexive responses such as physiological reactions (e.g., heart rate, skin-conductance, skin-temperature, respiration patterns, *etc*.) can be easily gauged through commercially available biomonitoring hardware and software. Alternatively, more complex high-level responses (e.g., actions, movements, speech, *etc*.) can be measured through a variety of different methods such as audio/video recordings of individuals as they experience different virtual environments. Moreover, these response data can be collected simultaneously enabling scientists to develop a more acute understanding of how various controlled environmental stimuli simultaneously affect multiple response systems.

As with all data collection methods, there are limitations to the use of IVEs. The first is cybersickness, which is nausea, dizziness and general discomfort caused by the unique stimuli of a virtual environment [22]. Cybersickness is experienced by a small fraction of individuals involved in IVE-based research, with almost all negative side effects dissipating after several minutes. Most individuals susceptible to cybersickness are aware they become nauseous when they do not have control of their movements (e.g., from previous experiences getting car- or sea-sick). These individuals tend to self-select out of participating in IVE-based research. However, all IVE-based research must require explicit informed consent about the possibility of becoming nauseous following individual institutions’ institutional review board guidelines. A second limitation is that developing IVE applications capable of collecting preference, perception and behavioral response data does require some familiarity with programming. This is not a major limitation however, as several IVE software packages come with detailed supporting documentation and have active online discussion groups. The third concern is the potential effect of the technology itself on biasing responses. Comparative research [23,24] has found different methods of displaying virtual environments elicits different emotional, perceptual and behavioral responses, raising concerns about the use of IVEs as the sole-method in which hypotheses are tested. This concern is valid; the findings generated from IVE-based research should be corroborated against other methods whenever possible. The next section illustrates how IVE-based research can complement traditional human perception, preference and behavior research focused on understanding how individuals perceive and respond to built and natural environments; an emphasis is placed on potential applications to public health research.

## 3. IVE Research as a Complement to Traditional Human Perception, Preference and Behavior Research

The suite of data collection methods available to social scientists interested in how humans perceive and respond to built and natural settings has increased over the past several decades. The dominant methods of representing built and natural environments has transitioned from the use of static imagery to the use of basic dynamic media (e.g., videos), to the use of interactive media (e.g., navigable walkthroughs) and finally to the use of fully immersive virtual simulations [25,26,27,28]. The transition from static to dynamic imagery illustrates a shift in scientists’ desire to move beyond describing social phenomenon (observational designs of individuals in the field) to discerning significant relationships (correlation analysis between specific image attributes and a variety of user characteristics) and eventually testing causal hypotheses by controlling variables of interest (experimental manipulation of image attributes). However, the shift to the use of dynamic imagery has also come at the expense of ecological validity (the realistic representation of an environment) as most early dynamic representations were created with computer hardware and software systems only capable of generating relatively simple and unrealistic virtual environments [29]. This trade-off between experimental control and ecological validity has been described as a major methodological problem facing psychological and behavioral research [30]. IVEs coupled with advances in computer technology (particularly processing speeds and graphic display capabilities) and software applications, however, provide the opportunity to both exert strict control over independent variables/treatments *and* present experimental stimuli in an extremely realistic format [1], thus providing the highly desirable ability to maximize internal validity while minimizing threats to external and construct validity. In short, IVEs provide scientists with the ability to maximize the benefits of traditional lab-based experiments (*i.e.*, control over independent variables and randomization of treatments) and field-based experiments (*i.e.*, high psychological realism of the phenomenon under study).

Several social science disciplines have capitalized on the development and recent advancements in IVE systems. The fields of psychology [2] and social psychology [1] have been using IVEs to address basic psychological and social phenomenon for several decades [31]. However, more applied disciplines such as environmental psychology [32] have been slower to integrate IVEs as a complementary methodological tool. Within the public health domain, substantive advances have been made in several specific areas such as obesity prevention and maintenance [33], dementia [34], genomics research [35] and health communication [36]. Within genomics research, Persky and McBride [35] outline how IVE technology could be used to assess and improving genetic test uptake rates amongst potential test-takers; however, the technology has yet to be applied in this context. Persky and McBride [35] also suggest IVE technology can be used to simulate distal consequences of individuals’ immediate decisions, a concept that was recently examined by Ahn [37] who found individuals exposed to a distal health outcome (weight gain as a result of soft drink consumption) through an IVE consumed less than individuals who only received information about the likely long-term effects of increased soft drink consumption.

Specific to behavioral research within the public health domain, Persky and McBride [35] suggest IVE technology provides an opportunity to develop patient-specific behavioral change interventions. Behavioral change appeals are most effective when tailored to patients’ mental states, health needs and sociodemographic characteristics [38] and IVEs provide an opportunity to rapidly modify select aspects of appeals (e.g., different modes of presenting health information), consequently improving the likelihood of achieving positive behavioral outcomes. Recent research within this area has yielded insightful findings. For example, Persky and her colleagues examined African-American patients’ ability to accurately describe risks associated with lung cancer after an IVE-based intervention in which they interacted with a virtual physician [39]. Risk perceptions were significantly less accurate when the patient interacted with a Caucasian as opposed to African-American physician; the effect was still significant even after controlling for patients’ perceived level of trust in the physician.

While the work of Persky and her colleagues is a focused example of using IVE technology to study behavioral change interventions, similar insight could be gained in other public health research that more directly involves assessing how individuals perceive and respond to built and natural settings. For example, health facilities around the United States have been rapidly adopting “park prescriptions”, which involve encouraging patients as well as members of the general public to use public park and recreation areas for physical activity in an effort to improve mental health and prevent chronic disease. Michelle Obama’s well known “Let’s Move Outside” campaign, the “Leave No Child Inside” campaign, the Children and Nature Network and the National Park Service’s “Healthy Parks, Healthy People” initiative are all notable examples. Despite the popularity of these campaigns and initiatives, no behavioral change research has examined whether or not they have altered individuals’ likelihood of visiting nearby parks and greenspaces. IVE-based research can be used to assess the efficacy of information disseminated by these programs amongst different populations. A variety of factors known to influence the adoption of preventive behaviors [38] such as the mode of information delivery (e.g., print *vs.* radio *vs.* television), the source of the information (e.g., physicians, celebrities, *etc*.) and the financial cost of adopting preventive behaviors are all likely to affect intended park use. Additionally, the quality and characteristics of available parks will influence individuals’ willingness to use them [40]. This diverse combination of factors, as well as others, has made it difficult for behavioral scientists to make definitive and empirically grounded statements about how best to promote the use of public parks and greenspaces amongst diverse publics. IVE technology provides the opportunity to examine and isolate confounding factors in a rigorous, yet flexible way. Different modes and sources of information can be examined across individuals with different sociodemographic backgrounds; and different park and neighborhood characteristics can be manipulated through the construction of different virtual environments. Hundreds of local, state and national campaigns, initiatives and programs have been developed to mitigate rising rates of chronic preventable diseases. However, these efforts almost always lack empirically grounded evaluative research that could improve their efficacy. IVE technology holds the promise of alleviating many of the methodological barriers faced by researchers in this area. In the subsequent sections, a description is provided of how social scientists can use IVEs as a supplement to traditional data collection methodologies. The discussion focuses on lab-based experiments and field-based experiments.

### 3.1. Traditional Lab-Based Experiments

Laboratory-based experiments are a particularly attractive methodology because they provide precise control over treatment effects. Investigators have direct control over both who is exposed to certain independent variables which allows randomization and control over the range across which those independent variables fluctuate. The ability to control independent (treatment) variables without affecting other confounding effects results in research findings with a high degree of internal validity [30]. The benefits derived from direct control over the administration of, and variability within, the independent variable, however, comes at the expense of external validity. Results from lab-based experiments are often criticized for their limited generalizability to large populations which is a major barrier to generating actionable policy and management recommendations [41]. Research attempting to understand preferences and perceptions associated with imagery of built and natural settings has typically been designed using lab-based experiments that present research participants with static color or grayscale images of either photorealistic scenes or computer generated illustrations [27,42]. This type of experimental research has focused primarily on measuring landscapes’ aesthetic appeal and using images portraying natural or semi-natural landscapes [27].

While the use of static color or grayscale images of landscapes in lab-based experiments has become relatively common, the approach does have several methodological shortcomings. Primarily, the mundane realism of lab-based experiments tends to be low, which means there is little concordance between how an individual experiences a particular treatment in the lab and how (or even if) they would experience it in their everyday lives [43]. Consequently, there is a concern the psychological processes (e.g., perceptions of safety) differ when a treatment is administered in the lab relative to when it is experienced in individuals’ everyday lives. The extent to which a specific psychological process can be reproduced in the lab setting is referred to as psychological realism [43]. IVEs can enhance the psychological realism of an experiment measuring human perceptions of, and preferences for, built and natural settings by supplementing existing lab-based methods. Consider for example, the growing body of research on the perceived safety of built and natural environments [44,45,46]. This scholarship has been built upon experimental methods whereby individuals are presented with a series of photographs, computer-generated renderings or illustrations, and asked to rate the images on rating scales (from extremely safe to extremely threatening). The images presented to participants are experimentally varied relative to some conceptually important independent variable. Typically, the independent variables include measures of an area’s enclosure or spaciousness. For example, a barrier such as a wall or hedgerow might be manipulated in its height and horizontal area. While this scholarship has made inroads into understanding how elements of the physical environment affect perceived safety, it is unclear whether the psychological process of fear-elicitation that individuals experience in day-to-day life can be comparably induced simply by having them look at a photograph, computer generated rendering or illustration. This could be a substantial threat to the validity of the research and clear analogs could be drawn to other domains of interest. The use of IVE technology can potentially mitigate the issue of low psychological realism given it allows various physical environmental stimuli to be presented in a highly realistic manner.

Applied psychology and social psychology research in other domain areas has already begun to capitalize on the high psychological realism IVE technology enables. For example, psychotherapists have successfully used virtual environments in the treatment of anxiety [47], eating disorders [48] and various phobias [49]. In short, supplementing controlled experimental designs with data generated through exposure to IVEs can increase the external validity of research findings due to IVEs increased capacity to present built and natural settings with an extremely high degree of mundane and psychological realism.

### 3.2. Field-Based Experiments

Given the criticisms of lab-based experiments’ failure to generate generalizable results, many investigators have opted to use field-based experimental designs. While field-based experiments offer the obvious advantage of high-levels of psychological realism, it is often extremely difficult to exert control over exogenous factors that might influence the relationship between the independent variable being manipulated across field settings and the dependent variable of interest.

As an example of the methodological difficulties inherent with field-based experiments, consider recent work using on-site behavior mapping [50], which involves the systematic observation and classification of individuals’ behavior relative to their spatial location within a physical environment. The method, which is gaining acceptance as a tool for mapping and monitoring visitor use in a variety of outdoor settings such as parks [51], allows investigators to discern how different environmental settings (e.g., meadows *vs.* forest edges) affect individuals’ behavioral patterns. Although research administrators follow strict observational protocols (e.g., times at which observations are recorded, the direction in which an environment is scanned) for recording individuals’ behavior, the primary methodological limitation of this approach is the inability to precisely determine what characteristics of an environment are responsible for eliciting certain responses from individuals; this is because these factors are difficult, if not impossible, to control [50,52]. Because of this threat to internal validity, behavior mapping research is often limited to demonstrating correlational findings, which of course are less persuasive in affecting policy change and enhancing scientific understanding.

Supplementing participant observation research with research using IVE technology offers a solution to the aforementioned methodological problem. IVE technology provides the ability to confirm and refine social and behavioral phenomenon observed in the field because investigators are able to evaluate the extent to which different physical characteristics elicit variations in behavioral responses. IVE-based research provides investigators with the ability to manipulate characteristics of built and natural settings that, in the field, are permanent or very difficult and costly to change. In a relatively short amount of time, an investigator can construct two or more nearly identical virtual environments with only one distinguishable factor. The behaviors of individuals within an immersion system could then be collected to discern more acutely and with a greater degree of internal validity the causal mechanisms driving the variations observed in the field settings.

The flexibility offered by virtual environments extends beyond modifications to the inanimate objects that comprise a built or natural setting. Researchers can also modify the characteristics and number of people present within those settings. This is a particularly appealing option for investigators studying how the use of a physical space affects individual perceptions and behaviors. For example, there is now a large literature devoted to understanding how the sociodemographic characteristics of existing park users affects potential park visitors’ willingness to use the space [53]. This research, however, has been impeded by an inability to manipulate the sociodemographic characteristics of park users and evaluate the subsequent perceptions and behaviors of *potential* park users [54]. IVE technology overcomes this impediment by providing investigators the ability to populate park areas with avatars of different races and ethnicities. IVE technology infinitely expands the ‘field’ in which traditional field-based research has been grounded. Mixed-methods approaches that use both IVEs and traditional field-based approaches allow investigators to explore contingent perceptions and behaviors that can be validated against reality.

Another advantage of IVE technologies in field-based and participant observation studies is its ability to simultaneously collect a variety of human responses that vary in ontological complexity. Eye tracking systems can measure simple or involuntary responses to virtual environments such as individuals’ gaze patterns and physiological attention measures such as blink-rate and pupil size [55]. Head tracking systems can measure slightly more complex responses, namely what objects individuals choose to orient their heads toward in a simulated environment [56,57]. Hand tracking devices can be used to collect data on even more complex responses such as what individuals choose to interact with in a virtual setting. Finally, and perhaps most interestingly, microphones synched to video recordings of individuals’ exploration of a virtual environment can be used to collect verbal responses from research participants [58]. Furthermore, IVE technology has the ability to incorporate qualitative methods into research designs to generate more detailed, and perhaps more conceptually insightful, findings to explain why individuals are engaging in a particular behavior, which is a major limitation of field-based research [59].

In brief, IVE technology has much to offer scientists with an interest in acutely measuring individuals’ perceptions of, preferences for, and behavioral responses to built and natural settings; the benefits are particularly salient for public health research. Specifically, IVEs can be used as a supplemental methodology to reduce or eliminate the limitations associated with lab-based and field-based experimental research designs as they can reproduce ‘real-world’ stimuli, duplicate psychological processes that occur in people’s daily lives, produce generalizable findings and generate more acute and internally valid measures of individuals’ preferences for, perceptions of, and behavioral responses to, built and natural settings.

## 4. Workflow

**Figure 1:**
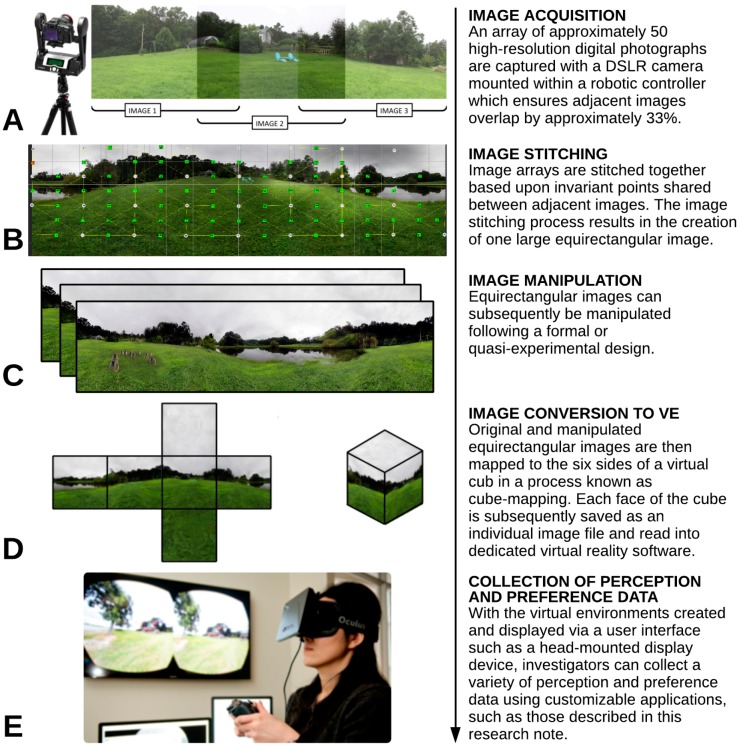
Workflow for developing immersive virtual environments of built and natural settings.

The construction of IVEs for research applications is a relatively simple and logical process requiring only a working knowledge of either digital image manipulation and processing or computer programming and application development. The workflow presented in Figure 1 can be used to guide readers’ understanding of IVE and application development. All of the workflow processes described use low-cost and commercially available hardware and software. The workflow follows a flexible five-step process involving: (1) image acquisition; (2) image stitching; (3) image manipulation; (4) image conversion to a virtual environment; and (5) data collection.

### 4.1. Image Acquisition

The workflow is initiated through the collection of digital imagery. One method for image acquisition involves a DSLR camera fitted within a robotic controller (e.g., GigaPan’s EPIC Pro controller). High quality DSLR cameras can be purchased for under $800 USD and the cost of robotic controllers does not exceed $1000 USD. The controller is mounted atop a tripod (Figure 1a) and precisely controls the movement of the camera, enabling it to be rotated around a single point. The controller is programmed with the camera’s field of view, which varies depending upon the camera’s zoom extent. The controller rotates the camera and triggers the shutter to collect an array of images. Each image overlaps adjacent images by approximately 33%. The image acquisition process culminates with the collection of an array of images that can be arranged in a rectangular grid.

### 4.2. Image Stitching 

The next step of the workflow involves stitching the rectangular array of images to create a full 360° equirectangular image with vertical dimensions that include both poles. The development of advanced image matching algorithms designed to detect and match invariant features appearing in image pairs (Figure 1b) has led to the creation of fully automated image stitching processes integrated into commercially available software; most software only requires the user to arrange images in their correct relative positions. The author’s lab group uses the Autopano Giga 4 image-stitching software produced by Kolor (cost is approximately $225 USD), however, other alternatives include PTGui Pro and Adobe Photoshop. Photo manipulation software can be used to correct inconsistencies in exposure, color balance and white levels. Once an image array is stitched together, it can be reverse projected and displayed as an IVE (discussed later). Custom curved mirrors attached to the camera lens can also be used to circumvent the need to stitch together image arrays; however, acquiring images in this way requires specialized software to re-project the single image for display via a navigable interface.

### 4.3. Image Manipulation 

This stage in the workflow process is optional; its inclusion will depend on the research questions being explored. The process involves taking an individual stitched equirectangular image and manipulating certain features within the image following an experimental design. While this stage is optional, it eliminates the possibility of confounding effects arising from the lack of complete experimental control. For example, photo-elicitation methods in environmental psychology have involved examining preferences for natural settings with varying biophysical attributes (e.g., preferences for forests that are either clear cut or selectively harvested). The photos used for comparison are often taken at different locations, at different times of day and contain potentially confounding features, such as the presence of rivers and wildlife, which might influence perception and preference data.

Image manipulation can follow any experimental design. The researcher only has to choose which attributes of the environment are of interest (e.g., extent of canopy coverage, presence of built features, *etc*.) that can be manipulated across a meaningful range. Following an experimental design, a series of manipulated images can be created; these image sets form the experimental treatments across which perception, preference and behavioral response data are collected (Figure 1c).

Image manipulation requires the use of photo editing software such as Adobe Photoshop (cost is approximately $300 USD) and should be done with high regard for maintaining realism [25]. A final image set should be consistent in ecological validity (*i.e*., respondents should not be able to discern which images are of artificially created environments). The author’s lab group pilot tests using online marketplaces (e.g., Amazon.com’s Mechanical Turk) where randomized image sets are presented to study participants who are asked to rank the set relative to the realism of each image. The rank scores are used to gauge degree of realism [60] which is compared between original and manipulated images using the Friedman non-parametric test. Image sets exhibiting significant variation in realism are rejected from inclusion in the final experimental design.

### 4.4. Image Conversion to Virtual Environment 

Converting stitched 2D equirectangular images into virtual environments involves a process known as cube mapping; this process uses a six-sided cube as a map shape [61,62]. The panoramic image is projected onto the cube’s faces; each face in turn is saved as an individual image file that will later be used to render the virtual environment (Figure 1d). Cube mapping can be completed through commercially available software; the author’s lab group uses Pano2VR (Garden Gnome Software).

The final step in displaying IVEs involves dedicated virtual reality software capable of rendering a virtual environment and displaying it in a user interface. The author’s lab group uses a commercially available software toolkit (Vizard; costs range from approximately $80 USD for the ‘Lite’ edition which is suitable for the applications described later to more than $10,000 USD for multi-seat ‘Development’ and ‘Enterprise’ editions) to read-in and map the six cube images and display the virtual environment in a user interface. The Vizard toolkit offers a high degree of flexibility in virtual environment creation, allows for the integration of multiple methods of interacting with virtual environments, and offers a low-cost edition.

### 4.5. Collection of Perception, Preference and Behavior Data

The final stage in the workflow involves the collection of data from research participants (Figure 1e). As noted above, IVE technology provides scientists with the capacity to collect an array of data varying in ontological complexity. Low-level responses such as physiological reactions (e.g., heart rate, skin-conductance, skin-temperature, respiration patterns, *etc*.) can easily be gauged through biomonitoring hardware and software. Higher level responses such as actions, movements and speech can be measured through various methods such as audio recordings of individuals’ responses to specific prompts. Response data can also be collected simultaneously, enabling scientists to develop a more acute understanding of how different controlled environmental stimuli simultaneously affect multiple behavioral response systems.

Collectively, the workflow is a relatively simple process for designing and developing virtual environments. In the final section of this paper, two freely available and modifiable custom applications created to gain a more empirically robust and valid understanding of how individuals perceive and respond to built and natural settings are presented.

## 5. IVE Applications for Environmental Preference and Behavior Research

**Figure 2:**
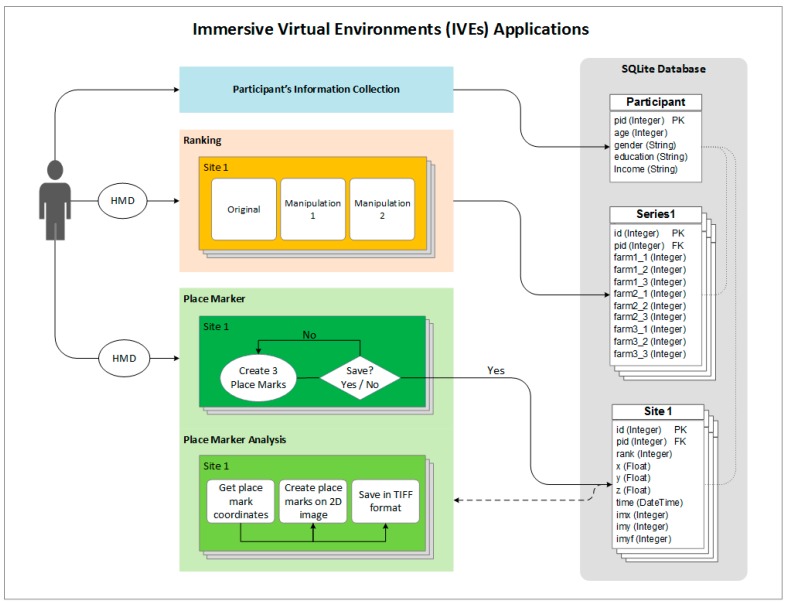
Workflow diagram for both the ranking and place marker applications (HMD = Head Mounted Display).

The first application involves generating ranking data from individuals’ preferences among three virtual environments. The second application generates Cartesian coordinates for markers placed by participants while experiencing a virtual environment. The process of implementing research designs using both of these applications is presented in Figure 2 and more detail is available at [63].

### 5.1. Ranking Application

Several well-developed lines of research are devoted to understanding and accurately gauging the “quality” of built and natural settings. Typically experts or members of a specific audience are queried about how the composition of a setting (*i.e*., presence, absence, or composition of biophysical features) affect its aesthetic appeal [64]. Given pictures elicit greater recollection and cognitive elaboration [65], two-dimensional images of built and natural settings are commonly used to query individuals’ perceptions on the aesthetic appeal, degree of realism, desirability or some other specific dependent measure related to those images. The first IVE-based application offers a more sophisticated approach, allowing individuals to rank virtual environments while experiencing them via a head mounted display.

As described in the workflow above, investigators first need to collect imagery at a minimum of three locations, stitch, manipulate (if following an experimental design) and convert images into virtual environments using cube-mapping software. Then, cube face imagery can be placed in a hierarchical file structure on a central, local machine. The application will construct three distinct virtual environments using the appropriate cube faces placed within this directory. The three constructed virtual environments comprise the choice-set that individuals are presented with while in a head mounted display.

The data collection process begins by reading a script to research participants detailing the task of ranking the environments (e.g., prompting individuals to rank the environments from “most safe” to “least safe”). Once instructions have been given, participants are placed in the head mounted display and asked to slowly and deliberately explore each of the virtual environments by moving their head. Participants only experience one environment at a time, but can directly compare the attributes of alternative environments through the use of a wireless game controller using the up and down buttons (This could alternatively be accomplished through keyboard command. However, the use of keyboards requires research participants to remain seated throughout the experiment, which is likely to lead to an inability to see and explore portions of the image behind the default viewing position). Once respondents have viewed and explored each virtual environment, they are prompted to select the most appealing environment with the aid of a wireless gaming remote. The application records a respondent’s initial selection in the database (e.g., *environment 3 = rank 1*) and removes that environment from the choice set. The respondent is again prompted to select between the remaining two environments and their subsequent responses are recorded accordingly in the database (e.g., *environment 2 = rank 2, environment 1 = rank 3*). This ranking experiment can be repeated as many or as few times as needed according to the experimental design being employed. The application’s sample code repeats the ranking process four times resulting in the collection of 12 data-points (four locations with three environments each; Table 1). These data can subsequently be analyzed using a variety of statistical techniques to discern significant variations across rankings within a single choice-set; they can also be analyzed using multi-level ranked logistic regression models to discern significant relationships across all choice sets.

**Table 1:** Example of data collected through the ranking application.

Location	Virtual Environment	Ranked Appeal/Preference Data	Analysis	
A	1 (original)	2		Ranked Logistic Regression
	2 (manipulation)	1		
	3 (manipulation)	3		
B	1 (original)	1	Friedman’s Rank Test	
	2 (manipulation)	3		
	3 (manipulation)	2		
C	1 (original)	3		
	2 (manipulation)	2		
	3 (manipulation)	1		
D	1 (original)	2		
	2 (manipulation)	1		
	3 (manipulation)	3		

### 5.2. Point Identification Application

Investigators focused on examining individuals’ perceptions, preferences and behaviors relative to different stimuli have a long-standing interest in discerning which characteristics of a built and natural setting elicit certain responses. For example, emerging work has used ocular tracking technologies, which map the focus of individuals’ gaze across a static image, to discern what characteristics of natural settings they focus on when viewing an image [66]. The second application presented here allows investigators to collect data on preferences for specific elements within virtual environments of built and natural settings (e.g., the presence, absence, or composition of biophysical features). Specifically, the application allows individuals to select specific points within a virtual environment after being given a prompt. Point locations are recorded by the application, thus providing investigators the ability to generate highly acute maps corresponding to individuals’ preferences. Again, a sample executable and detailed description of the application is available at [63].

**Figure 3:**
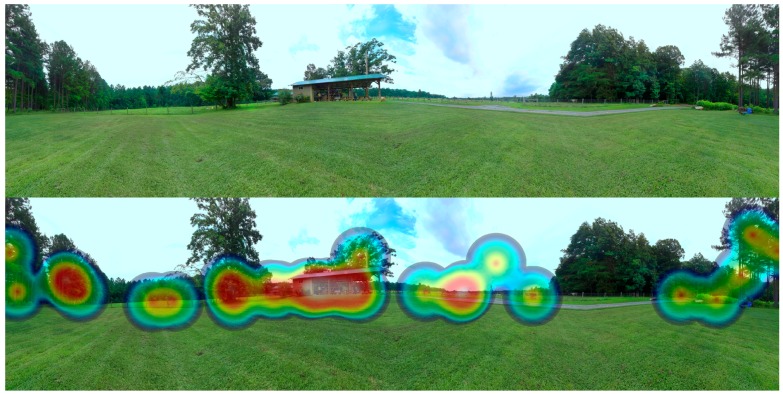
Example of an unfolded virtual environment without (**above**) and with (**below**) a heat map visualization of points generated with the place marker application.

The point identification application uses a singular virtual environment, which can either represent a real or digitally manipulated setting. After receiving specific instructions, participants are asked to explore the virtual environment and locate specific “points” (which correspond to the variables being examined) with a target-like icon originally placed at the center of their field of vision. For example, individuals can be prompted to identify three points within the environment that deter from its use as a place for physical activity. Participants confirm their selections with a wireless gaming controller. The application’s sample code version allows individuals to identify three specific points within the environment. Points can be elicited through prompts that vary relative to the specific research question being investigated. The application records both the world (spherical) and environment (cube-mapped) coordinates of respondents’ point selections in the database. The collection of these coordinates provides the ability to disassemble the cube into its six sides and retain the location of user-defined points. The Cartesian (two-dimensional) coordinates can subsequently be visualized relative to their concentration (Figure 3 provides an example). Beyond visualization, investigators can generate summary statistics of points located in specific areas of a setting or explore the data with more advanced spatial statistics.

## 6. Conclusions

IVE technology is quickly becoming a ubiquitous part of our modern technologically integrated lives. The widespread availability of IVE technology has made it an additional methodological tool accessible to investigators studying human perceptions, preferences and responses to built and natural settings. This article has detailed how IVE technology can be used to supplement traditional methods focused on discerning how human perceptions, preferences and behaviors change relative to varying environmental stimuli; particular emphasis has been placed on possible avenues for future research within the public health domain. Robust mixed-methods designs using IVE technology along with more traditional lab- or field-based experiments hold the potential to contribute not only to methodological rigor, but also to the examination of novel and previously unexplored phenomena. Several specific research areas where IVE technology could have an immediate impact have been described. Specifically, perception, preference and behavior research has typically used data collected by presenting static imagery to individuals in a lab environment or via web-based or paper questionnaires. This methodology, while being foundational to our understanding of environmental preferences and perceptions, is limited in its ability to realistically and wholly replicate environments as individuals experience them in their everyday lives. IVE technology holds the promise of bolstering this shortcoming through the generation of virtual environments that can be experienced and explored with nearly as much realism as daily life [67].

Other field-based methodologies, such as behavior mapping also have potential to be augmented with IVE technologies. Field-based experimental designs can be easily criticized for their lack of internal validity and inability to control for exogenous factors that may confound the relationship between environmental characteristics and perception, preference or behavioral responses. IVE technologies provide these field-based investigations with the opportunity to wholly replicate built and natural settings save for specific features believed to influence the dependent variable of interest.

This article also presented a workflow for creating and displaying 360° virtual environments of built and natural settings. The workflow is not intended to be a step-by-step guide for adopting IVE technology. Rather, it is intended to be sufficiently detailed enough to provide individual investigators and research teams with direction and guidance on how the use of IVE technology can be initiated. Further development and refinement will begin with investigators’ individual needs and capabilities. To further assist scientists in their potential adoption of IVE technology, sample code for two freely-available and editable applications has been developed and presented. The first application collects ranking data from individuals’ preferences among three virtual environments while the second collects coordinate locations of points placed by users as they experience and explore a virtual environment. Both applications have the potential to be used in the near future to address a wide variety of research questions.

In conclusion, the use of IVE technology has become more and more common in applied arenas of certain fields such as medicine, psychotherapy and education. However, the technology’s use to address long-standing research questions related to human perceptions of, and preferences for, built and natural settings has remained limited. Due to increasing consumer demand, immersion systems are well within the reach of nearly all researchers. The description of the technology’s advantages as a research method, its workflow and sample applications for capturing and displaying built and natural settings, will enable more scientists to expand the boundaries of their work and develop a deeper understanding of the complex interactions between individuals and the environments in which they live.
